# Supplementary material for: Collateral Effect of the Coronavirus Disease 2019 Pandemic on Emergency Department Visits in Korea
Source: Medicina (Kaunas). 2022 Dec 31;59(1):90. doi: 10.3390/medicina59010090 (PMC9862451; doi:10.3390/medicina59010090)
Supplement: Supplementary file 1 [file medicina-59-00090-s001.zip › Supplementary Table S1.pdf]

Supplementary Table S1. Numbers of patients who visited an ED from 2018 to 2020 listed by diagnosis.

	LAD				SARI				AHS				AIS				AMI				CA			
Month	**2018**	**2019**	**2020**	**2020 (%)** [a]	**2018**	**2019**	**2020**	**2020 (%)** [a]	**2018**	**2019**	**2020**	**2020 (%)** [a]	**2018**	**2019**	**2020**	**2020 (%)** [a]	**2018**	**2019**	**2020**	**2020 (%)** [a]	**2018**	**2019**	**2020**	**2020 (%)** [a]
Jan	18662	14631	21544	129.42	16888	7314	16168	133.61	400	462	398	92.34	815	918	1030	118.87	436	453	492	110.69	305	327	362	114.56
Feb	17026	14315	10116	64.55	7754	3684	3391	59.29	362	393	393	104.11	732	826	825	105.91	432	397	440	106.15	285	296	289	99.48
Mar	14377	12927	5543	40.60	4023	5124	1248	27.29	398	474	405	92.89	817	962	844	94.88	435	436	399	91.62	262	323	314	107.35
Apr	14985	14168	5220	35.81	4512	8271	1025	16.04	381	434	424	104.05	859	977	878	95.64	472	443	448	97.92	249	298	324	118.46
May	16538	15299	7637	47.98	5296	5362	1278	23.98	367	420	398	101.14	916	1072	1008	101.41	457	471	489	105.39	294	274	325	114.44
Jun	15428	15024	7722	50.72	3411	3436	1349	39.40	368	359	362	99.59	823	998	982	107.85	417	436	446	104.57	226	290	286	110.85
Jul	17345	15124	8485	52.27	2559	2728	1154	43.65	347	343	352	102.03	927	988	1050	109.66	465	496	432	89.91	249	267	303	117.44
Aug	17151	15534	8724	53.38	2368	2777	1197	46.53	332	338	346	103.28	872	971	984	106.78	413	449	453	105.10	249	297	320	117.22
Sep	19298	15999	5518	31.27	3139	3739	1019	29.63	341	363	413	117.33	828	999	883	96.66	419	491	425	93.41	255	272	297	112.71
Oct	12608	11926	6824	55.63	3105	4036	1174	32.88	423	407	404	97.35	915	1018	1000	103.47	473	513	530	107.51	278	292	356	124.91
Nov	13405	11991	6182	48.68	5460	5069	1160	22.03	388	439	415	100.36	902	1033	1032	106.67	464	474	445	94.88	278	311	366	124.28

Dec	19741	17243	6177	33.40	19145	12739	1030	6.46	446	449	396	88.49	951	1030	952	96.11	468	535	457	91.13	361	364	373	102.90
Total	196564	174181	99692	53.78	77660	64279	31193	43.95	4553	4881	4706	99.77	10357	11792	11468	103.55	5351	5594	5456	99.70	3291	3611	3915	113.45

[a] For the investigation of monthly trends from 2018 to 2020, the monthly numbers of ED visits in 2020 were compared with the average numbers of visits of the corresponding months in 2018 and 2019.

ED = emergency department, LAD = low-acuity disease, SARI = severe acute respiratory infection, AHS = acute hemorrhagic stroke, AIS = acute ischemic stroke, AMI = acute myocardial infarction, CA = cardiac arrest.
