# Supplementary material for: Collateral Effect of the Coronavirus Disease 2019 Pandemic on Emergency Department Visits in Korea
Source: Medicina (Kaunas). 2022 Dec 31;59(1):90. doi: 10.3390/medicina59010090 (PMC9862451; doi:10.3390/medicina59010090)
Supplement: Supplementary file 1 [file medicina-59-00090-s001.zip › Supplementary Table S2.pdf]

Supplementary Table S2. Deaths within 7 days of an ED visit from 2018 to 2020 listed by diagnosis.

	LAD				SARI				AHS				AIS				AMI				CA			
Month	**2018**	**2019**	**2020**	**2020 (%)** [a]	**2018**	**2019**	**2020**	**2020 (%)** [a]	**2018**	**2019**	**2020**	**2020 (%)** [a]	**2018**	**2019**	**2020**	**2020 (%)** [a]	**2018**	**2019**	**2020**	**2020 (%)** [a]	**2018**	**2019**	**2020**	**2020 (%)** [a]
Jan	5	10	9	120.00	144	103	119	96.36	44	51	33	69.47	15	21	24	133.33	33	25	31	106.90	266	296	308	109.61
Feb	5	2	8	228.57	84	90	84	96.55	41	47	45	102.27	12	16	22	157.14	37	33	37	105.71	251	262	242	94.35
Mar	7	4	5	90.91	88	90	64	71.91	39	42	45	111.11	12	26	25	131.58	38	18	40	142.86	225	279	282	111.90
Apr	7	2	2	44.44	65	79	72	100.00	30	45	40	106.67	29	17	22	95.65	17	38	24	87.27	220	260	273	113.75
May	4	6	5	100.00	87	76	75	92.02	44	37	51	125.93	18	27	34	151.11	40	33	35	95.89	244	238	286	118.67
Jun	5	6	4	72.73	66	66	69	104.55	45	32	40	103.90	8	15	15	130.43	35	29	27	84.38	195	248	248	111.96
Jul	3	3	9	300.00	74	73	73	99.32	39	27	33	100.00	13	16	24	165.52	31	32	36	114.29	213	236	264	117.59
Aug	8	10	8	88.89	62	59	64	105.79	31	33	44	137.50	18	13	15	96.77	25	32	30	105.26	211	263	288	121.52
Sep	12	5	4	47.06	63	60	80	130.08	36	39	43	114.67	18	19	20	108.11	28	37	27	83.08	207	235	257	116.29
Oct	4	6	5	100.00	67	83	73	97.33	34	42	48	126.32	24	20	21	95.45	33	30	41	130.16	240	256	320	129.03
Nov	6	1	6	171.43	75	77	80	105.26	44	36	43	107.50	22	27	12	48.98	28	34	29	93.55	243	264	321	126.63
Dec	3	3	6	200.00	104	75	82	91.62	39	59	41	83.67	22	14	24	133.33	29	30	27	91.53	318	326	330	102.48

Total	69	58	71	111.81	979	931	935	97.91	466	490	506	105.86	211	231	258	116.74	374	371	384	103.09	2833	3163	3419	114.04
(fatality rate, %)[b]	(0.04)	(0.03)	(0.07)		(1.26)	(1.45)	(3.00)		(10.24)	(10.04)	(10.75)		(2.04)	(1.96)	(2.25)		(6.99)	(6.63)	(7.04)		(86.08)	(87.59)	(87.33)	

ED = emergency department, LAD = low-acuity disease, SARI = severe acute respiratory infection, AHS = acute hemorrhagic stroke, AIS = acute ischemic stroke, AMI = acute myocardial infarction, CA = cardiac arrest.

[a]For the investigation of monthly trends from 2018 to 2020, the 7-day deaths rates after ED visits for each month in 2020 were compared with the average numbers of the corresponding months in the control period.

[b]Fatality rate = 100 × number of deaths within 7 days of an emergency department visit ÷ number of patients who visited an emergency department
