# Supplementary material for: Collateral Effect of the Coronavirus Disease 2019 Pandemic on Emergency Department Visits in Korea
Source: Medicina (Kaunas). 2022 Dec 31;59(1):90. doi: 10.3390/medicina59010090 (PMC9862451; doi:10.3390/medicina59010090)
Supplement: Supplementary file 1 [file medicina-59-00090-s001.zip › Supplementary Table S3.pdf]

Supplementary Table S3. Monthly incidence of ED visits by age in 2020 compared to the average incidence during the control period.

	LAD				SARI				AHS				AIS			AMI			CA			
	<18	18–75	>75	Total	<18	18–75	>75	Total	<18	18–75	>75	Total	18–75	>75	Total	18–75	>75	Total	<18	18–75	>75	Total
Jan	120.57	134.23	124.93	129.40	140.90	140.35	94.13	133.60	133.33	90.91	95.47	92.30	125.72	110.69	118.90	114.19	103.70	110.70	93.33	117.68	112.11	114.60
Feb	48.84	73.18	58.10	64.60	46.60	63.90	73.33	59.30	66.67	105.00	103.06	104.10	107.18	104.37	105.90	119.35	78.97	106.20	166.67	99.08	98.39	99.50
Mar	22.44	50.91	47.74	40.60	8.33	34.17	59.13	27.30	66.67	94.61	89.45	92.90	107.64	79.60	94.90	90.42	94.69	91.60	83.33	107.50	108.30	107.40
Apr	18.43	47.93	50.31	35.80	3.49	21.98	53.38	16.00	200.00	103.89	103.60	104.00	104.32	85.88	95.60	103.02	84.92	97.90	66.67	129.54	108.17	118.50
May	27.00	62.89	61.11	48.00	8.78	28.52	55.35	24.00	80.00	106.98	87.00	101.10	104.30	97.69	101.40	111.22	92.88	105.40	114.29	122.82	104.94	114.40
Jun	30.84	63.19	68.11	50.70	16.72	46.50	71.17	39.40	114.29	103.46	89.00	99.60	112.91	101.58	107.90	107.18	98.81	104.60	77.78	124.11	96.30	110.90
Jul	31.28	65.70	64.81	52.30	17.67	52.47	69.93	43.70	160.00	109.81	82.52	102.00	120.30	96.74	109.70	91.94	85.22	89.90	155.56	130.80	98.17	117.40
Aug	32.45	64.88	69.48	53.40	17.02	53.46	76.56	46.50	80.00	106.35	94.41	103.30	118.45	91.07	106.80	113.17	85.37	105.10	285.71	117.73	111.67	117.20
Sep	17.34	39.02	45.90	31.30	6.80	36.29	77.05	29.60	133.33	113.16	128.04	117.30	101.36	90.95	96.70	100.00	77.94	93.40	160.00	112.50	110.80	112.70
Oct	35.46	66.61	76.89	55.60	12.32	37.20	75.85	32.90	80.00	90.58	117.70	97.30	114.63	89.30	103.50	108.51	105.12	107.50	80.00	142.07	108.15	124.90
Nov	30.26	60.49	64.15	48.70	9.48	26.10	65.76	22.00	250.00	111.30	72.58	100.40	107.47	105.14	106.70	100.31	82.88	94.90	153.85	136.77	108.27	124.30
Dec	21.03	40.59	47.57	33.40	2.23	6.05	39.76	6.50	114.29	92.33	77.12	88.50	95.91	96.24	96.10	96.93	76.66	91.10	28.57	111.41	97.86	102.90
Total	35.28	64.79	65.35	53.80	27.50	52.87	68.89	44.00	117.24	101.80	94.21	99.80	109.84	95.64	103.60	104.34	89.01	99.70	106.57	120.43	105.29	113.40

ED = emergency department, LAD = low-acuity disease, SARI = severe acute respiratory infection, AHS = acute hemorrhagic stroke, AIS = acute ischemic stroke, AMI = acute myocardial infarction, CA = cardiac arrest.
