# Supplementary material for: Collateral Effect of the Coronavirus Disease 2019 Pandemic on Emergency Department Visits in Korea
Source: Medicina (Kaunas). 2022 Dec 31;59(1):90. doi: 10.3390/medicina59010090 (PMC9862451; doi:10.3390/medicina59010090)
Supplement: Supplementary file 1 [file medicina-59-00090-s001.zip › Supplementary Table S4.pdf]

Supplementary Table S4. Monthly incidence of ED visits by region in 2020 compared with that during the control period.

	LAD				SARI				AHS				AIS				AMI				CA			
Month	**SKI**	**DK**	**Other**	**Total**	**SKI**	**DK**	**Other**	**Total**	**SKI**	**DK**	**Other**	**Total**	**SKI**	**DK**	**Other**	**Total**	**SKI**	**DK**	**Other**	**Total**	**SKI**	**DK**	**Other**	**Total**
Jan	126.02	123.96	133.70	129.40	146.66	141.82	122.16	133.60	86.67	90.32	98.68	92.30	120.27	97.70	123.50	118.90	108.66	113.21	111.94	110.70	105.84	86.02	137.66	114.60
Feb	61.03	60.86	68.53	64.60	51.29	75.61	62.88	59.30	89.89	91.95	126.03	104.10	98.93	97.35	114.61	105.90	99.73	76.92	122.90	106.20	97.73	120.00	96.03	99.50
Mar	38.57	27.61	45.32	40.60	24.62	29.41	29.61	27.30	105.85	81.40	82.44	92.90	93.08	67.32	103.78	94.90	94.79	81.74	91.56	91.60	112.88	111.76	100.40	107.40
Apr	32.35	28.48	40.68	35.80	13.68	18.74	17.85	16.00	99.73	118.52	105.26	104.00	103.06	86.83	91.36	95.60	99.00	82.24	100.99	97.90	129.27	112.12	108.94	118.50
May	42.42	42.18	54.80	48.00	20.97	21.95	27.37	24.00	107.65	114.29	91.43	101.10	112.34	85.48	96.33	101.40	106.50	100.00	105.74	105.40	107.98	133.33	116.32	114.40
Jun	45.61	45.20	57.07	50.70	35.76	45.89	41.63	39.40	100.90	91.36	100.32	99.60	114.10	100.00	104.32	107.90	96.86	98.15	114.60	104.60	96.53	130.91	123.76	110.90
Jul	48.79	50.72	56.26	52.30	38.80	49.10	47.15	43.70	112.70	106.85	90.32	102.00	108.08	100.00	113.82	109.70	101.88	116.67	75.61	89.90	121.55	113.85	114.16	117.40
Aug	46.28	55.49	60.15	53.40	45.68	56.06	45.32	46.50	103.43	85.00	108.55	103.30	111.17	126.47	98.14	106.80	102.44	103.51	108.18	105.10	124.91	141.94	100.47	117.20
Sep	28.65	32.72	33.43	31.30	26.82	47.39	28.90	29.60	123.38	110.00	113.29	117.30	99.73	83.62	97.56	96.70	99.46	95.50	87.59	93.40	108.80	98.55	122.12	112.70
Oct	50.20	59.35	59.97	55.60	29.88	41.60	33.93	32.90	96.28	96.77	98.61	97.30	106.36	87.70	105.17	103.50	109.22	133.96	99.78	107.50	119.57	110.77	135.71	124.90
Nov	41.87	51.61	54.82	48.70	17.70	26.87	26.25	22.00	97.65	114.63	100.00	100.40	106.86	120.95	103.19	106.70	85.78	101.03	103.52	94.90	116.54	117.50	134.98	124.30
Dec	30.21	33.38	36.28	33.40	6.22	9.04	6.19	6.50	85.05	115.79	86.77	88.50	98.94	78.11	98.10	96.10	92.31	82.54	92.41	91.10	114.81	121.95	85.89	102.90
**Total**	49.37	51.33	58.55	53.80	41.33	51.26	44.94	44.00	100.07	101.00	99.15	99.80	106.11	93.90	103.70	103.60	99.55	98.10	100.26	99.70	112.90	115.31	113.52	113.40

ED = emergency department, LAD = low-acuity disease, SARI = severe acute respiratory infection, AHS = acute hemorrhagic stroke, AIS = acute ischemic stroke, AMI = acute myocardial infarction, CA = cardiac arrest, SKI = Seoul, Gyeonggi, Incheon, DK = Daegu, Gyeongbuk.
