# Supplementary material for: Collateral Effect of the Coronavirus Disease 2019 Pandemic on Emergency Department Visits in Korea
Source: Medicina (Kaunas). 2022 Dec 31;59(1):90. doi: 10.3390/medicina59010090 (PMC9862451; doi:10.3390/medicina59010090)
Supplement: Supplementary file 1 [file medicina-59-00090-s001.zip › Supplementary Table S5.pdf]

Supplementary Table S5. Changes in incidence of ED visits by regions in 2020 compared to the average incidence during the control period

	LAD			SARI			AHS			AIS			AMI			CA		
	SKI	DK	Others	SKI	DK	Others	SKI	DK	Others	SKI	DK	Others	SKI	DK	Others	SKI	DK	Others
Prepandemic	126.02	123.96	133.70	146.66	141.82	122.16	86.67	90.32	98.68	120.27	97.70	123.50	108.66	113.21	111.94	105.84	86.02	137.66
1st wave	44.41	39.49	52.22	28.77	41.22	37.42	98.60	96.85	102.49	98.26	83.47	102.43	97.88	80.24	104.38	112.92	114.57	101.62
2nd wave	37.31	43.54	46.04	34.92	51.30	35.83	113.20	97.50	111.11	105.61	103.67	97.85	100.94	99.56	97.27	117.15	119.08	111.11
3rd wave	35.12	40.52	43.64	9.35	13.43	10.69	91.31	115.19	92.81	102.82	98.42	100.67	89.04	90.58	97.72	115.59	119.75	107.12
Total wave[b]	39.53	41.02	47.84	20.06	29.23	24.49	100.00	102.10	101.40	101.75	93.91	100.56	95.87	88.69	100.32	114.92	117.48	105.74
Inter-wave	46.54	48.90	56.87	29.62	36.47	35.73	104.02	102.11	95.20	110.12	93.00	104.67	103.80	111.90	97.46	111.30	121.91	122.40

The first wave occurred between February and April 2020, the second wave occurred between August and September 2020, and the third wave lasted from November to the end of December 2020. We defined January as prepandemic, May, Jun, July, and October as inter-wave.

[a]ED visit change (%) = 200 × ED visit of 2020 / (ED visit of 2018 + ED visit of 2019) – 100.

[b]Total wave : February, March, April, August, September, November, and December.

ED = emergency department, LAD = low-acuity disease, SARI = severe acute respiratory infection, AHS = acute hemorrhagic stroke, AIS = acute ischemic stroke, AMI = acute myocardial infarction, CA = cardiac arrest, SKI = Seoul, Gyeonggi, Incheon, DK = Daegu, Gyeongbuk.
