# Supplementary material for: Collateral Effect of the Coronavirus Disease 2019 Pandemic on Emergency Department Visits in Korea
Source: Medicina (Kaunas). 2022 Dec 31;59(1):90. doi: 10.3390/medicina59010090 (PMC9862451; doi:10.3390/medicina59010090)
Supplement: Supplementary file 1 [file medicina-59-00090-s001.zip › Supplementary Table S6.pdf]

Supplementary Table S6. Numbers of ED visits, admissions, and deaths reported in the 2018–2020 Korea's NEDIS Statistical Yearbook.

	2018			2019			2020			2020 (%)[a]		
Month	ED visit	Admission	Death	ED visit	Admission	Death	ED visit	Admission	Death	ED visit	Admission	Death
Jan	947642	155294	3497	794858	141403	2942	989252	148771	3041	113.54	100.28	94.46
Feb	829757	136186	2690	783579	128723	2502	588177	109869	2621	72.91	82.95	100.96
Mar	775150	144775	2619	796636	143950	2663	484388	101626	2947	61.64	70.40	111.59
Apr	811016	148728	2553	831465	147945	2488	522147	108328	2845	63.58	73.03	112.87
May	914103	160051	2427	887702	156896	2536	660296	126286	2621	73.29	79.69	105.62
Jun	875896	153664	2342	865981	150878	2342	660798	126821	2524	75.87	83.29	107.77
Jul	920812	160795	2432	863355	155495	2268	669655	130665	2480	75.07	82.62	105.53
Aug	915196	157944	2469	917287	157202	2407	710669	125366	2732	77.56	79.56	112.06
Sep	993695	152978	2365	959331	151801	2363	596689	113583	2638	61.10	74.53	111.59
Oct	824647	149901	2765	837581	149637	2551	704557	125960	3100	84.77	84.10	116.63
Nov	771730	146387	2554	772817	144483	2782	580219	116992	2888	75.13	80.44	108.25
Dec	1006440	157773	3076	906686	151131	2824	523464	109283	3208	54.72	70.76	108.75
total	10586084	1824476	31789	10217278	1779544	30668	7690311	1443550	33645	73.93	80.11	107.74

[a]For the investigation of monthly trends from 2018 to 2020, the monthly numbers of 2020 were compared with the average numbers of 2018 and 2019.

ED = emergency department, NEDIS = National Emergency Department Information System.
