# Supplementary figures and images for: Collateral Effect of the Coronavirus Disease 2019 Pandemic on Emergency Department Visits in Korea
Source: Medicina (Kaunas). 2022 Dec 31;59(1):90. doi: 10.3390/medicina59010090 (PMC9862451; doi:10.3390/medicina59010090)

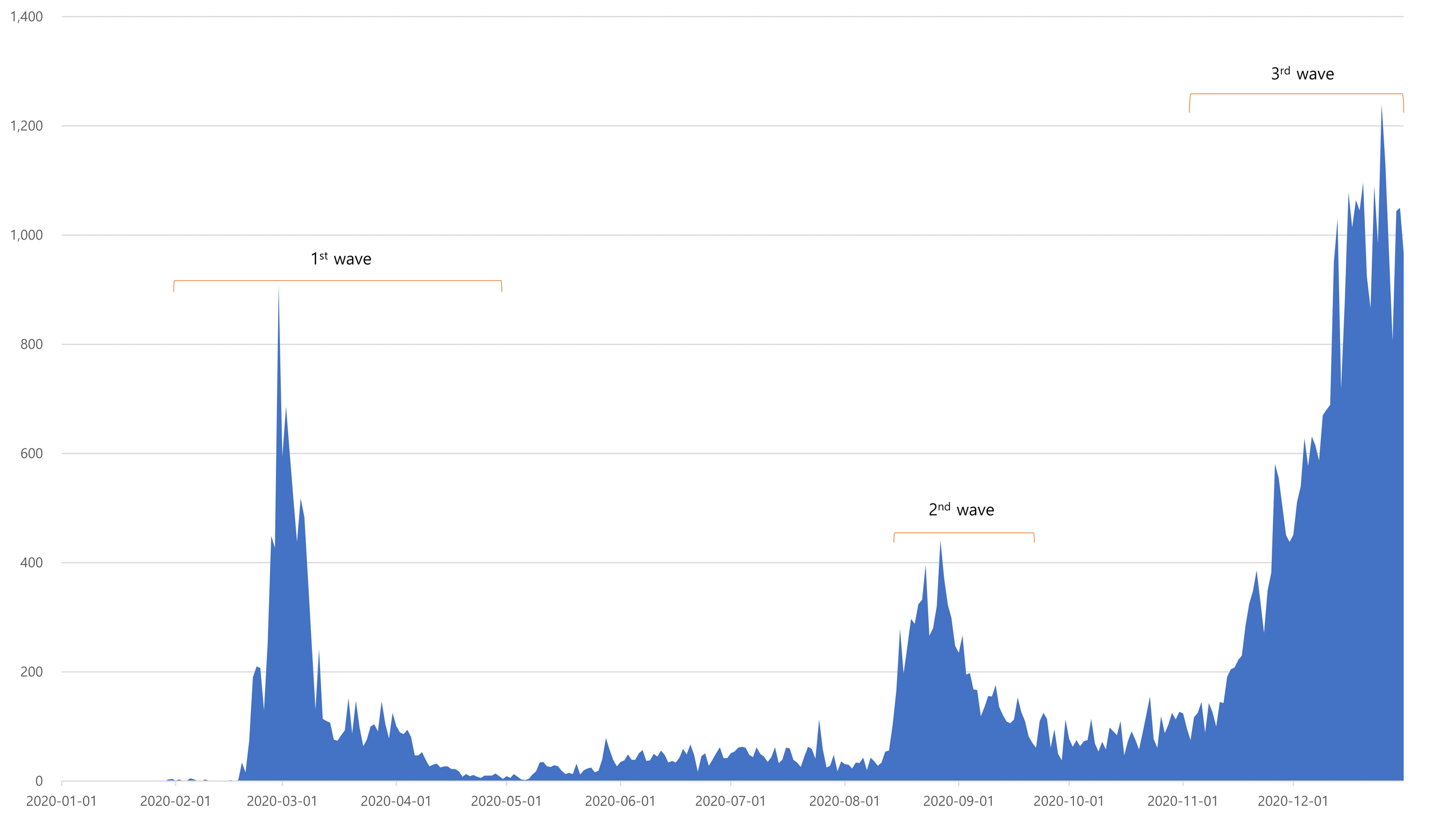

Supplement: Supplementary file 1 [file medicina-59-00090-s001.zip › supplement Figure S1.PNG]

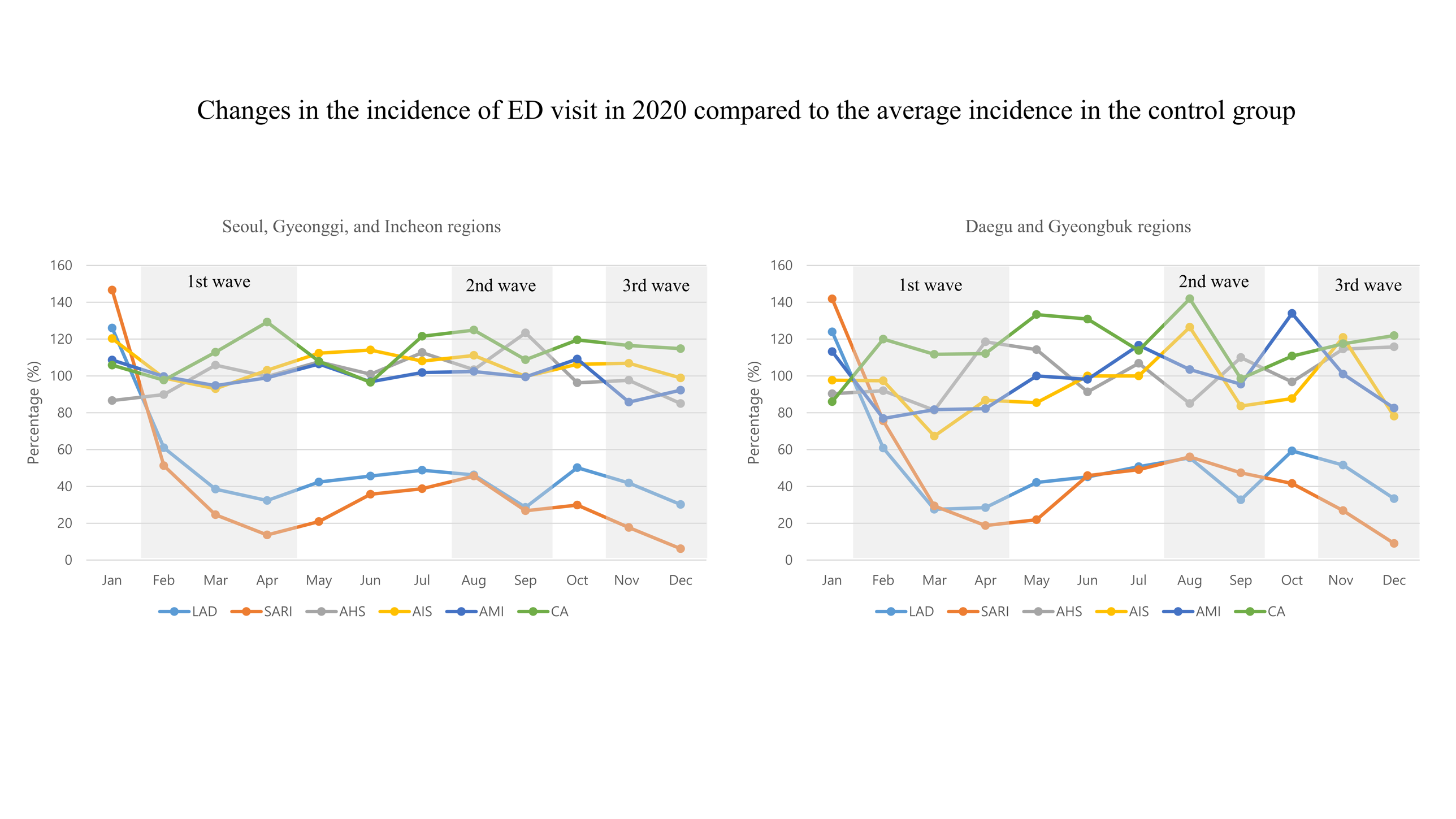

Supplement: Supplementary file 1 [file medicina-59-00090-s001.zip › supplement Figure S2.png]
